# Chemically driven energetic molecular ferroelectrics

**DOI:** 10.1038/s41467-021-26007-2

**Published:** 2021-09-29

**Authors:** Yong Hu, Zhiyu Liu, Chi-Chin Wu, Jennifer L. Gottfried, Rose Pesce-Rodriguez, Scott D. Walck, Peter W. Chung, Shenqiang Ren

**Affiliations:** 1 Department of Mechanical and Aerospace Engineering, University at Buffalo, The State University of New York, Buffalo, NY 14260 USA; 2 Department of Mechanical Engineering, University of Maryland, College Park, MD 20740 USA; 3 Weapons and Materials Research Directorate, U.S. Army Research Laboratory, Aberdeen Proving Ground, Aberdeen, MD 21005 USA; 4 Research and Education in Energy Environment & Water Institute, University at Buffalo, The State University of New York, Buffalo, NY 14260 USA; 5 Department of Chemistry, University at Buffalo, The State University of New York, Buffalo, NY 14260 USA

**Keywords:** Devices for energy harvesting, Ferroelectrics and multiferroics

## Abstract

Chemically driven thermal wave triggers high energy release rate in covalently-bonded molecular energetic materials. Molecular ferroelectrics bridge thermal wave and electrical energy by pyroelectric associated with heating frequency, thermal mass and heat transfer. Herein we design energetic molecular ferroelectrics consisting of imidazolium cations (energetic ion) and perchlorate anions (oxidizer), and describe its thermal wave energy conversion with a specific power of 1.8 kW kg^−1^. Such a molecular ferroelectric crystal shows an estimated detonation velocity of 7.20 ± 0.27 km s^−1^ comparable to trinitrotoluene and hexanitrostilbene. A polarization-dependent heat transfer and specific power suggests the role of electron-phonon interaction in tuning energy density of energetic molecular ferroelectrics. These findings represent a class of molecular ferroelectric energetic compounds for emerging energy applications demanding high power density.

## Abstract (web summary)

An interest arises if energetic molecules and molecular ferroelectrics can be integrated together to obtain a chemically driven electricity conversion. Here, the authors explore energetic molecular ferroelectrics consisting of imidazolium cations and perchlorate anions with a high-power density.

## Introduction

**Fig. 1:**
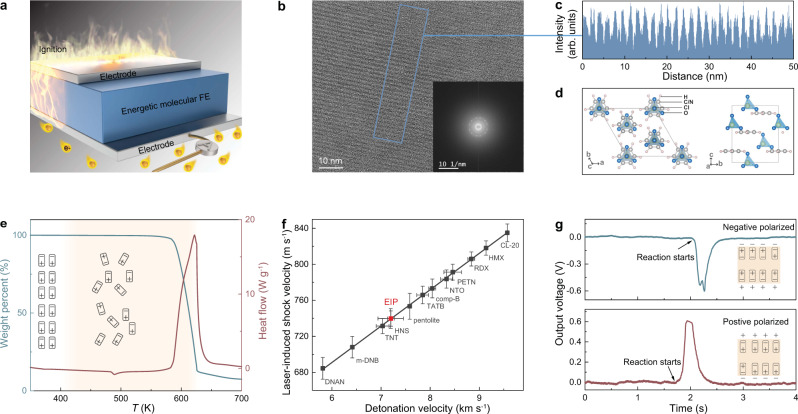
Chemical driven electricity in EIP. **a** Schematic figure for electricity generation in energetic molecular ferroelectrics (FE). **b** HRTEM images for EIP. Inset shows the FFT pattern reflecting the high crystallinity of EIP. **c** Line profile for selected area in Fig. 1b. **d** Crystal structure of ferroelectric EIP. **e** Thermogravimetric analysis and differential scanning calorimetry measurement for EIP. Supplementary Figure 5a highlights the transition at 373 K. **f** The laser-induced shock and detonation velocity of EIP. Error bars show standard deviations. DNAN (2,4-dinitroanisole); *m*-DNB (*m*-dinitrobenzene); TNT (trinitrotoluene); HNS (hexanitrostilbene); TATB (triaminotrinitrobenzene); comp-B (Composition B); NTO (5-nitro-2,4-dihydro-3H-1,2,4-triazole-3-One); PETN (pentaerythritol tetranitrate); RDX (cyclotrimethylenetrinitramine); HMX (cyclotetramethylene tetranitramine); CL-20 (hexanitrohexaazaisowurtzitane). **g** The DC voltage generated by reaction of EIP is observed immediately after ignition in both positive and negative polarization. EIP devices are ignited thermally. The gap distance between the electrodes is 1.2 mm and the cross-sectional area is 16 mm^2^.

Molecular crystals constructed by the C–N, N–N, and N–O bonds store chemical energy, while its strong electron–phonon coupling interactions promise high-energy density through thermal waves^1,2^. At the same time, symmetry breaking in molecular crystals induces self-polarization and electron–phonon interaction, leading to molecular ferroelectrics^3,4^. An interest arises if these two dissimilar materials (molecular energetic materials and ferroelectrics) can be integrated together to obtain a chemically driven electrical energy with high-power density that can be employed for emerging technological applications, e.g. an on-demand energy source, propulsion, or thermal battery. Here we describe how a large chemically driven specific power could be obtained in an energetic molecular ferroelectric crystal showing strong electron-phonon interactions. Figure 1a and Supplementary Fig. 1 show the schematic diagrams for the chemically driven energy generator using energetic molecular ferroelectrics. Electrons and dipoles are electrostatically coupled at the ferroelectrics/electrode interface due to the spontaneous polarization. The large specific thermal energy release of molecular ferroelectrics could cause thermal and shock waves which propagate and induce electricity through the pyroelectric effect in ferroelectrics^5–8^.

To integrate energetic property and spontaneous polarization in a molecular crystal, design of a delicate molecular system is necessary. To create a molecular ferroelectric chemical generator, we surmise the following three design parameters: (1) macromolecular ferroelectrics with a high critical temperature, *T*_c_; (2) simultaneous qualities of high-energy density of chemical bonds and high-energy release rate; and (3) a large pyroelectric coefficient for high specific power. We select energetic imidazolium perchlorate (EIP) molecular crystal as the chemically driven energy generator candidate due to its energetic bonds and high ferroelectric performance, among molecular ferroelectrics with reported high *T*_c_ (Supplementary Table S1)^9–12^. The imidazolium cation (C_3_H_5_N_2_^+^) and perchlorate anion (ClO_4_^−^) have been successfully demonstrated as important components in energetic materials^13,14^. The unique properties of C_3_H_5_N_2_^+^ cation stem from the electronic structure of the aromatic cations which is characterized by 3-center-4-electron configuration across the N_1_–C_2_–N_3_ bonds, and a double bond between C_4_ and C_5_ (Supplementary Fig. 2). Oxygen is another important ingredient of energetic materials which is required to convert carbon into oxocarbons and hydrogen into water. The lack of oxygen in C_3_H_5_N_2_^+^ can be overcome by using a suitable oxidizer ClO_4_^−13^. The EIP shows a large pyroelectric coefficient of −6334 μC m^−2^ K^−1^. An estimated detonation velocity of 7.20 ± 0.27 km s^−1^ based on a characteristic laser-induced shock velocity of 740.0 ± 11.1 m s^−1^, with a high-power density up to 1.8 kW kg^−1^, is obtained in EIP, which is comparable to trinitrotoluene (TNT) and hexanitrostilbene (HNS).

## Results

The high-resolution transmission electron micrograph (HRTEM) shows pronounced fringes from a thin region of ferroelectric EIP (Fig. 1b). The average inter-spacing of these fringes are calculated as 1.699 nm from the line profile acquired by a selected area (Fig. 1c). In general, the high crystalline nature of EIP is also demonstrated by the strong diffraction contrast, as shown in Supplementary Fig. 3. The EIP shows a non-centrosymmetric space group R3m at 298 K (Fig. 1d) and a centrosymmetric space group R$\bar{3}$m at 385 K (Supplementary Fig. 4)^9,10^. In both phases the C_3_H_5_N_2_^+^ rings are located at the 3m symmetry site perpendicular to the threefold axis. The perchlorate anions behave differently, which are well ordered in the non-centrosymmetric phase and disordered in the centrosymmetric phase^9,10^. Three transitions of EIP are observed in the differential scanning calorimetry (DSC, Fig. 1e). The first transition at 373 K results from the second-order ferroelectric–paraelectric phase transition with a ∼1.2 kJ kg^−1^ heat absorption^9–11^. The second transition at 489 K is a first-order transition^10^ along with a relatively large heat absorption ∼22.8 kJ g^−1^. The third transition at 622 K, with large weight loss indicating phase decomposition with bond breaking, brings about a large energetic performance (Fig. 1f). The EIP undergoes disproportionation reaction at 622 K with an ∼87% mass loss and a large ∼3066.6 kJ kg^−1^ heat release under N_2_, suggesting its energetic nature. It has to be mentioned that a larger heat release of 3810.6 kJ kg^−1^ is obtained under air which provides additional oxygen for the reaction (Supplementary Fig. 5b). The decomposition temperature is relatively high, avoiding undesirable decomposition or self-initiation during their handling, storing, and applications. In addition, the lower transition temperature from ferroelectric to paraelectric phase paves the way for the continuous electricity generation.

The chemically driven electricity is studied by building EIP devices (Fig. 1a), where the EIP is polarized by a saturated electric field (12 kV cm^−1^) before all measurements. As shown in Fig. 1g, a voltage pulse with the peak of 0.61 V is obtained during ignition when the device is positively polarized. The pulse width is around 0.4 s, suggesting the fast heating and decomposition of EIP, which is favorable for obtaining large pyroelectric current according to the equation:

1 $${i}_{\mathrm p}={pA}\frac{{{\mathrm d}T}}{{{\mathrm d}t}}$$

where ${i}_{\mathrm p}$ is the pyroelectric current, d*T*/d*t* is the rate of temperature change, *A* is the surface area of material, and *p* is the pyroelectric coefficient. It has to be mentioned that the polarity of the measured voltage is highly related to the polarization direction. When the pre-polarizing direction is reserved, a voltage pulse with a peak of −0.60 V is obtained, suggesting the origin of pyroelectric effect. In addition, the control experiment shows that chemically driven electricity cannot be achieved in the paraelectric phase (Supplementary Fig. 6). This chemically driven pyroelectric effect is distinct from conventional pyroelectric harvesters, in which the frequency of thermal fluctuation is limited to a low value (typically <1 Hz) due to its large thermal mass and thermal inertia. The results suggest that chemically driven charge release is faster than that of a thermally induced effect. Such a fast charge release could result in a higher specific power, which will be discussed in the later section. To understand the chemically driven electricity generation, we perform further studies on the dielectric property and energetic behavior of EIP.

**Fig. 2:**
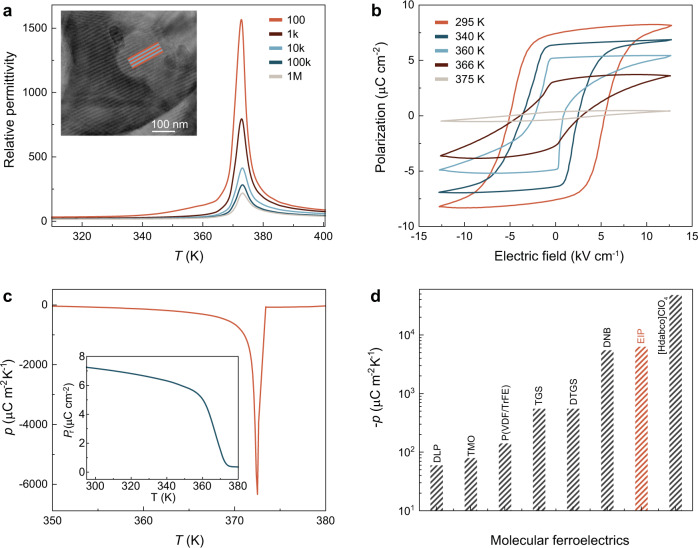
Ferroelectric and pyroelectric properties of EIP. **a** Temperature dependence of relative permittivity for EIP at different frequencies. Inset shows a HRTEM for unpoled EIP. **b** *P*–*E* loops of EIP measured at 200 Hz and different temperatures. **c** Temperature-dependent pyroelectric coefficient (*p*) for EIP. Inset shows remnant polarization as a function of temperature. **d** Pyroelectric coefficients for representative molecular ferroelectrics with EIP highlighted in red.

Figure 2a presents the temperature dependence of relative permittivity of EIP at different frequencies, which shows the characteristic sharp dielectric anomalies around the ferroelectric–paraelectric phase transition temperature of 373 K. Inset of Fig. 2a shows the HRTEM image for ferroelectric EIP with distinct moiré fringes, suggesting its self-polarization induced ferroelectric domain structures^8,12^. The ferroelectric nature of EIP is further confirmed by the current–electric field (*I*–*E*) curve. As shown in Supplementary Fig. 7, the current is maximized exactly at the coercive field instead of the maximum electric field, providing direct evidence for its ferroelectricity. We further carry out the polarization–electric field (*P*–*E*) loops at different temperatures. As shown in Fig. 2b, at 295 K, a coercive field of 5.4 kV cm^−1^ and a large saturated polarization (*P*_s_) of ~8.2 μC cm^−2^ is attained, which is favorable to obtain a large pyroelectric coefficient. After increasing temperature, the hysteresis loops become slim and the *P*_s_ is reduced. The temperature-dependent remnant polarization (*P*_r_) is shown in the inset of Fig. 2c. The *P*_r_ is less than *P*_s_ as mechanical boundary conditions normally impose direction changes in some domains while removing the electric field. The *P*_r_ changes continuously but not abruptly at around *T*_c_, suggesting a second-order phase transition. The EIP only has one polar direction^9,12^, thus the total value of *p* along the polarization direction is^15^

2 $$p=\underbrace{\frac{\partial\, {{{{{\mathrm{Pr}}}}}}}{\partial T}}_{{p}_{prim}}+\underbrace{\mathop{\sum }_{i,j}\frac{\partial {d}_{ij}{\sigma }_{ij}}{\partial T}}_{{p}_{sec}}+\underbrace{\mathop{\sum }_{i,j,k}\partial μ_{i,j,k} \frac{\frac{\partial e_{i,j}}{\partial r_{k}}}{\partial T}}_{p_{tert}}$$

Mechanical stress (*σ*), position vector (*r*), piezoelectric tensor (*d*), flexoelectric tensor (*μ*), and strain tensor (*e*). The change of *P*_r_ during temperature fluctuations induces the primary pyroelectric effect (${p}_{{{{{\mathrm{prim}}}}}}$). The mechanical and thermal expansion induced strain cause the secondary pyroelectric effect (${p}_{{{{{\mathrm{sec }}}}}}$). The tertiary pyroelectric effect $({p}_{\mathrm {tert}})$ is due to spatial strain gradients, which can also be induced by deformation during non-uniform heating or cooling. The pyroelectric measurements are carried out using the Byer–Roundy method^16^, which mainly account for the primary and secondary pyroelectric effects due to the a slow heating rate of 5 K min^−1^. The maximum pyroelectric coefficient of −6,334 μC m^−2^ K^−1^ is achieved in EIP at the *T*_c_ of 373 K (Fig. 2c, d). The pyroelectric coefficient of EIP is comparable to inorganic ferroelectrics, like 0.68Pb(Mg_1/3_Nb_2/3_)O_3_−0.32PbTiO_3_ (−7300 μC m^−2^ K^−1^) and Ba_0.85_Ca_0.15_Zr_0.1_Ti_0.9_O_3_ (−980 μC m^−2^ K^−1^), and higher than ferroelectric polymers, like poly (vinylidene fluoride-trifluoroethylene) (−142 μC m^−2^ K^−1^)^17–19^. It should be mentioned that shock wave would be generated during the fast decomposition of EIP, which could induce tertiary pyroelectric effect.

**Fig. 3:**
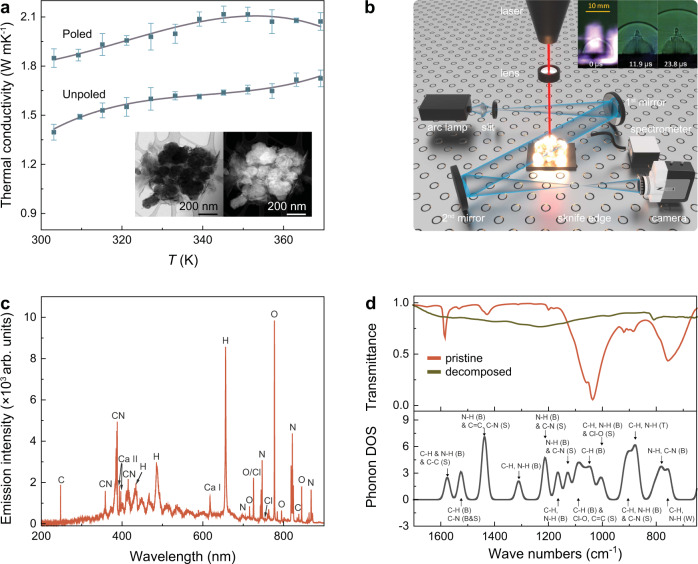
Thermal and energetic performance of EIP. **a** Temperature dependence of thermal conductivity for poled and unpoled EIP. Inset show the HRTEM for unpoled EIP. **b** Schematic figure for laser-induced shock velocity images obtained from high-speed video. Arc lamp generates light which is focused through a slit onto the first mirror. The collimated light between the first and second mirror detects the change in the refractive index of air above the sample surface. A knife edge is applied to reduce approximately half the light rays. A spectrometer is used to simultaneously measure the plasma emission spectrum. Inset shows the selected snapshots from the high-speed video for laser-shocked EIP. **c** Plasma emission spectrum for EIP during decomposition. The only impurity is Ca, and the only molecular recombination species observed during the first 10 μs is CN. **d** FTIR spectra for EIP before and after decomposition. Calculated phonon density of states (DOS) for EIP. bending (B); stretching (S); twisting (T); wagging (W). The phonon dispersion is shown in Supplementary Fig. 13.

The molecular EIP ferroelectrics show superior electromechanical coupling^9,12^ with flexible molecular chains^12,20^, suggesting the primary pyroelectric effect is mainly contributed by electronic redistribution caused by thermal vibrations (known as electron–phonon renormalization)^21,22^. Phonon is the predominant heat carrier in electrically insulating EIP, lying in the center of thermal conduction. Thermal conductivities of poled and unpoled EIP samples are measured to reveal the electron-phonon interaction (Fig. 3a). During the heating process, its thermal conductivity increases and then decreases approaching *T*_c_ due to the loss of polarization. Inset of Fig. 3a shows the bright field and high-angle annual dark-field STEM images of unpoled EIP. The dark-field STEM image not only corresponds excellently with bright field, but also reveals the details of different crystalline phases within the overall crystalline structure in unpoled EIP. In addition, the absence of moiré fringes for unpoled EIP indicates the change in the ferroelectric domain structures. For the unpoled state, its thermal conductivity is 1.39 W mK^−1^ at 303 K, ∼26% less than in the poled state which is mainly attributed to the dipole induced change of phonon-phonon scattering and ferroelectric domain structures^23–25^. Such polarization dependence of thermal conductivity suggests the interaction between phonon and electron in EIP, promising for its chemically driven electricity.

We further study chemical decomposition at high heating rates (~10^13^ K s^−1^) to understand the kinetics of the chemical energy release from EIP. The laser-induced air shock from energetic materials (LASEM) method is used to measure the microsecond timescale energy release (Fig. 3b)^26^. This measurement is based on high-speed schlieren imaging of the laser-induced shock wave that results when the EIP is ablated into a high-temperature plasma; the subsequent chemical reactions in the rapidly cooling plasma influence the velocity of the shock wave at times <10 μs, such that the more energetic the material is the faster the laser-induced shock wave. The characteristic laser-induced shock velocities for conventional energetic materials have been shown to directly correlate to the measured detonation velocities from large-scale testing^26^. Thus, the laser-induced shock velocity based on milligram amounts of material can be used to estimate the detonation velocity of the bulk material (assuming the material is detonable and could be pressed at the theoretical maximum density). The characteristic laser-induced shock velocity for EIP is 740.0 ± 11.1 m s^−1^, which gives an estimated detonation velocity of 7.20 ± 0.27 km s^−1^. The energetic performance of EIP is comparable to TNT and HNS (Fig. 1d). During the detonation of an explosive, the detonation shock wave passes through the bulk material-unlike in LASEM, where the shock wave passes through the plasma above the sample surface. The passage of the detonation wave through the bulk material containing EIP could potentially influence electrical conductivity of the reacting material^27^ and the subsequent large-scale detonation performance.

The plasma emission spectrum for EIP is measured during the LASEM experiments (averaged over 20 laser shots). As shown in Fig. 3c, the EIP decomposition produces Ca, C, CN, H, N, O, and Cl, where Ca is a minor impurity (likely from solvent contamination) and CN is formed via the recombination reactions during plasma cooling. The scanning electron microscopy (SEM, Supplementary Figs. 8 and 9) and energy dispersive spectroscopy (EDS) results (Supplementary Table S2) show that the decomposed EIP contains micron-sized pores and significantly reduced O and Cl elements, suggesting the gaseous products. We further performed the pyrolysis study for EIP at 623 K. As shown in Supplementary Fig. 10, the main products are permanent gases [*m*/*z* = 44 (CO_2_), *m*/*z* = 28 (CO or N_2_), *m*/*z*  = 43 (HNCO]) and much smaller amounts of imidazole-related species. The FTIR result shows the characteristic vibrational modes (Fig. 3d) for C–H, N–H bending modes (1584 cm^−1^); C–H bending and C–N bending and stretching modes (1531); N–H bending and C=C, C–N stretching modes (1428 cm^−1^); C–H bending and C–N bending and stretching modes (1312 cm^−1^); N–H bending mode (1200 cm^−1^); C–H bending mode (1036 cm^−1^); C–H, N–H twisting modes (883 cm^−1^); C–H, N–H wagging modes (757 cm^−1^). The characteristic vibrational modes are diminished in the decomposed phase, suggesting the associated bond breaking after decomposition.

**Fig. 4:**
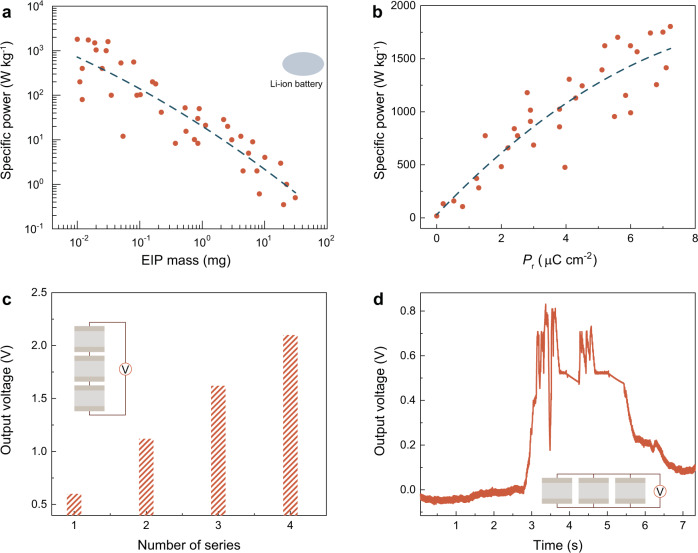
Chemical driven electricity performance in EIP. **a** The specific peak power plotted as a function of EIP mass. Two kinds of EIP devices are prepared. One is made by EIP crystals with sliver epoxy electrode. The other one is made by EIP thin films in the range of (1–100) μm on the prepatterned indium tin oxide substrate (50 μm channel length). **b** *P*_r_ dependence of specific power. **c** Enhanced output voltage achieved by series configuration circuit. **d** Extended working time by parallel configuration circuit. For devices in the series and parallel configuration circuit test, the gap distance between the electrodes is 1.2 mm and the cross-sectional area is 16 mm^2^ for each device.

The decomposition-induced thermal and shock wave during the detonation is favorable for inducing a fast temperature change to maximize the pyroelectric power in the EIP. Traditional chemically driven electricity shows a low specific power as it relies on a chemical potential-driven reaction which follows the rate law. In addition, a complex system is usually required to harvest chemical energy, such as a battery or a fuel cell. Examining the specific power as a function of mass (Fig. 4a) for 42 sample devices demonstrates a sample-to-sample variation, mostly resulting from the differences of the decomposition process. An optimum specific power in EIP is observed, as high as 1.8 kW kg^−1^. As the chemically driven pyroelectric effect is highly related with the remnant polarization, we further examine the specific power under different remnant polarization. As shown in Fig. 4b, the specific power shows an increased tendency as the remnant polarization increases. The output specific power of EIP is also subject to the load resistances. Supplementary Figure 14 shows that the output power shows a maximum level of 1.8 kW kg^−1^ under a 5.5 kohm load resistance which is about 150 times under a 200 kohm load resistance and about 500 times under a 5.6 Mohm load resistance. Figure 4d shows that the output voltage shows a tendency to increase as the number of devices is increased. A large output voltage of 2.1 V is obtained when four EIP devices are connected by the series configuration circuit. In addition, Fig. 4d shows that the short electricity generation duration can be overcome by adopting a parallel configuration circuit. A continuous electricity generation of 3 s could be achieved when six devices are connected in parallel.

We report two dissimilar materials, molecular energetic materials and ferroelectrics, can be integrated together to obtain a chemically driven electrical energy with a high specific power of 1.8 kW kg^−1^ and achieve an estimated detonation velocity of 7.20 ± 0.27 km s^−1^ comparable to TNT and HNS. Thermal analysis confirms that EIP releases a large thermal energy of 3810.6 kJ kg^−1^ at the decomposition temperature of 622 K. Polarization-dependent thermal conductivity and specific power reveal the important role of electron–phonon interaction in the chemically driven electricity through molecular energetic ferroelectrics consisting of covalently bonded cation fuel and anion oxidizer. A large thermal and shock wave from energetic compound decomposition results in a fast electrical energy release due to the pyroelectric effect in molecular ferroelectrics. Our studies reveal that this chemically driven energy generator is a result of the coupling between energetic thermal and shock waves and a pyroelectric effect in molecular ferroelectrics.

## Methods

### Synthesis of EIP

EIP crystals are synthesized by evaporating the aqueous solution of imidazole hydrochloride (Sigma-Aldrich) and perchloric acid (70%, Sigma-Aldrich) (molar ratio: imidazole hydrochloride/perchloric acid = 1/1). The EIP thin film devices are made by drop-casting method.

### Structure and elemental analysis

SEM and EDS measurements are performed by using a Hitachi S4000 SEM. An Agilent Cary 630 FTIR spectrometer is applied for FTIR measurement.

### Thermal analysis

Thermal analysis is carried out by a thermogravimetric analyzer (TGA) & DSC (DSC SDT Q600, TA Instruments, USA) under Air and N_2_ atmosphere at 10 K min^−1^. The thermal conductivities of EIP at different temperatures are measured by using a transient plane source sensor-based thermal conductivity analyzer (HOT-DISK TPS 2200).

### Pyroelectric, dielectric, and electrical characterization

The pyroelectric measurement is performed with Keithley 6512 electrometer at 5 K min^−1^. *P*–*E* loops and *I*–*E* curve are measured with a Radiant Ferroelectric Tester Precision LC. The probe is a Radiant High Voltage Cryogenic Probe. And the temperature control is realized by Quantum Design Physical Property Measurement System (PPMS). Temperature dependence of relative permittivity measurements are performed with an Agilent 4294A impedance analyzer in a PPMS. Electrical measurements are carried out by a Keithley 2450 SMU.

### LASEM measurements

Samples of as-received polarized and depolarized (via DSC heating and cooling) EIP are prepared for LASEM analysis by spreading a thin layer of approximately 20 mg of material on a piece of double-sided tape affixed to a glass microscope slide. The LASEM technique is performed as previously described^26^. Briefly, a 6-ns-pulsed laser (Quantel Brilliant b, 1064 nm, 850 mJ) is focused just below the sample surface to ablate micrograms of material and form a laser-induced plasma in the air above the sample, resulting in the atomization, ionization, and/or excitation of the ablated material. Most of the energy in the laser pulse is absorbed by the plasma, resulting in a heating rate on the order of 10^13^ K s^−1^. On the microsecond timescale, high-temperature chemical reactions of the excited material occur. These reactions influence the velocity of the laser-induced shock wave generated by the focused laser pulse as it passes through the plasma region (i.e., for approximately 7–9 μs depending on the sample). The shock wave position as a function of time is measured using a custom Matlab program based on high-speed schlieren videos obtained at a frame rate of 84,000 fps with a 1 μs shutter (Photron SA5 camera). Twenty laser shots are obtained for each sample. In addition, the plasma emission for each laser shot is collected by a reflective collimator based on a parabolic mirror with a UV-enhanced Al coating and delivered to an echelle/intensified charge-coupled device detector (Catalina Scientific SE200 with an Apogee camera, 200–1000 nm, 0.02-nm resolution, gain set to 890) via a fiber optic cable to produce emission spectra integrated over 10 μs (delayed by 1.5 μs to minimize the broadband continuum emission from excited electrons in the plasma).

### Pyrolysis study

Desorption and pyrolysis products are analyzed by means of a gas chromatography/mass spectrometry (GC/MS) instrument with a desorption interface. Desorption is achieved via a CDS Analytical Model 2000 Pyroprobe (coil type) connected through a heated interface chamber to the splitless injector of an Agilent (Santa Clara, California) GC/MS system (Model 6890N GC and Model 5973N MSD). The GC column used is a HP-5 capillary 250 column (0.25 mm × 30 m, 0.25-μm film). The injector temperature is 523 K; the Pyroprobe interface is set to a temperature of 523 K. The GC oven temperature program is as follows: 373 K isothermal for 1 min, 373–523 K at 40 K min^−1^, and 523 K isothermal for 1 min. The Pyroprobe is programmed to give a 20-s desorption pulse at temperatures of 175 and 523 K at a heating rate of 1000 K s^−1^. All analyses for a given carbon (C) sample type are run sequentially on a single sample. The pulse temperature is based on calibration provided by the vendor and is not measured for this study. Samples (approximately 1 mg) are held within the coil of the Pyroprobe by first placing them in a quartz tube containing a small plug of glass wool, and then inserting the entire tube into the coil.

### TEM analyses

The TEM specimens are prepared using acetone to suspend particles onto the holey carbon support film of TEM copper grids (300 mesh, Ted Pella, Inc.). The high-resolution bright field imaging and fast Fourier transform (FFT) pattern acquisition are performed in the TEM mode. Scanning mode TEM is also performed to acquire high-resolution bright field and high-angle annular dark-field images for heated EIP. All TEM images are analyzed using the Gatan Microscopy Suite Software (version 3, Gatan Inc.).

### DFT calculation

The structure of EIP is non-centrosymmetric with space group R3m at room temperature^10^. The C_3_H_5_N_2_^+^ rings are located at the 3m symmetry site perpendicular to the threefold axis. The rings are in the shape of pentagons composed of three carbon and two nitrogen atoms. The rings are disordered but their orientations are uniformly distributed as determined by the position of the apex carbon atom between the two nitrogen atoms. The apex atom of each ring can sit at one of six symmetrically equivalent positions. These positions can be indicated using the vertices of an underlying regular hexagon. The perchlorate anions are in the shape of tetrahedrons lying on the threefold axis and are well ordered. The DFT unit cell consists of three imidazolium cations and three perchlorate anions. To account for the effect of the disordered pentagons on the lattice structure, the orientations of the three pentagons are allowed to vary using two types of unit cell configurations. One orients the 3 pentagons arbitrarily, which leads to 10 unique configurations. The second approach restricts the apex carbons in the three rings to occupy three non-consecutive vertices of the regular hexagon. Overall, 15 variants of the unit cell structure are considered. After relaxing the structure using the procedure described further below, the unit cells constructed using the second approach appeared to match experimental lattice angles exactly. The overall lowest energy configuration is found to occur for a configuration with the apex carbon atom of the edge ring of the unit cell lying on the short diagonal of the rhombus.

The structural relaxation and phonon calculation are performed with the QUANTUM ESPRESSO code^28^. Ultrasoft pseudopotentials (USPP) with the generalized gradient approximation (PBE) form of the exchange-correlation functional is used. The vdW interaction is modeled using the semi-empirical correction proposed by Grimme (Grimme-D2)^29^. The reciprocal space is sampled with a 4 × 4 × 4 mesh, and the plane wave energy cutoff is chosen to be 60 Ry. Full ion relaxation is performed until the force acting on the atom is less than 1 × 10^−5^ Ry/Bohr and the energy difference is within 1 × 10^−6^ Ry. Phonon calculations are based on density functional perturbation theory (DFPT) as implemented in QUANTUM ESPRESSO. A 4 × 4 × 4 Monkhorst-Pack q point grid is used to obtain the dynamical matrices. The phonon density of states is obtained with a uniform 20 × 20 × 20 q-grid.

## Supplementary information

Supplementary Information

## Data Availability

All relevant data are included within this article and its Supplementary Information files. Any additional information is available from the corresponding author on reasonable request.
