# Supplementary material for: Chemically driven energetic molecular ferroelectrics
Source: Nat Commun. 2021 Sep 29;12:5696. doi: 10.1038/s41467-021-26007-2 (PMC8481480; doi:10.1038/s41467-021-26007-2)
Supplement: Supplementary file 1 — Supplementary Information [file 41467_2021_26007_MOESM1_ESM.pdf]

Supplementary Information for

Chemically Driven Energetic Molecular Ferroelectrics

Yong Hu[1], Zhiyu Liu[2], Chi-Chin Wu[3*], Jennifer L. Gottfried[3*], Rose Pesce-Rodriguez[3], Scott D Walck[3], Peter W Chung[2] & Shenqiang Ren[1,4,5*]

Affiliations:

[1]Department of Mechanical and Aerospace Engineering, University at Buffalo, The State University of New York, Buffalo, NY 14260, USA

[2]Department of Mechanical Engineering, University of Maryland, College Park, MD 20740, United States

[3]Weapons and Materials Research Directorate, U.S. Army Research Laboratory, Aberdeen Proving Ground, Aberdeen, MD 21005, USA

[4]Research and Education in Energy Environment & Water Institute, University at Buffalo, The State University of New York, Buffalo, NY 14260, USA

[5]Department of Chemistry, University at Buffalo, The State University of New York, Buffalo, NY 14260, USA

*Correspondence to: chi-chin.wu.civ@mail.mil; jennifer.l.gottfried.civ@mail.mil; shenren@buffalo.edu

Supplementary Table S1. Representative molecular ferroelectrics with high T_c.

	Molecular crystals	T_c (K)	Reference
1	Imidazolium Perchlorate （IM）	373	1
2	Triglycine sulfate	322	2
3	trimethylbromomethylammonium tribromomanganese(II) (TMBM-$MnBr_3$)	415	3
4	Guanidinium perchlorate	454	4
5	[Hdabco]ClO_4; Hdabco = monoprotonated 1,4-diazabicyclo[2.2.2]octane	377	5
6	[Hdabco]BF_4; Hdabco = monoprotonated 1,4-diazabicyclo[2.2.2]octane	374	6
7	[gua]ClO_4; gua = guanidinium	454	7
8	[Et_4N]ClO_4; Et_4N = tetraethylammonium	378	8
9	[qui]IO_4; qui = quinuclidinium	322	9
10	[hqu]Cl; hqu = (R)-(−)-3-hydroxlyquinuclidinium	340	10
11	[apd]$RbBr_3$; apd = 3-ammoniopyrrolidinium	440	11
12	[MeHdabco]RbI_3; MeHdabco = protonated N-methyl-1,4-diazoniabicyclo[2.2.2]octane	430	12
13	$[tmno]_2[KFe(CN)_6]$; tmno = protonated trimethylamine N-oxide	402	13
14	[tmcm]$MnCl_3$; tmcm = trimethylchloromethylammonium	406	14
15	Diisopropylammonium bromide	426	15
16	1-Azabicyclo[2.2.1]heptanium perrhenate	322	16
17	Poly(vinylidene fluoride), (PVDF)	363	17
18	PVDF–TrFE	373	17
19	diisopropylammonium bromide	425	18
20	tetramethylammonium tetrachloroferrate(III)	344	19
21	tetramethylammonium bromotrichloroferrate-(III)	346	19
22	tetramethylammonium	383	20

	tetrachlorogallate(III)		
23	Croconic acid	420	21
24	phenylmalonaldehyde (PhMDA)	363	21,22
25	5,6-dichloro-2-methyl-1H-benzimidazole (DC-MBI)	399	21,23
26	2-methyl-1Hbenzimidazole (MBI)	430	21,23
27	Triglycine sulfate (TGS)	323	24
28	3-hydroxy-1H-phenalen-1-one (HPLN)	420	21,22

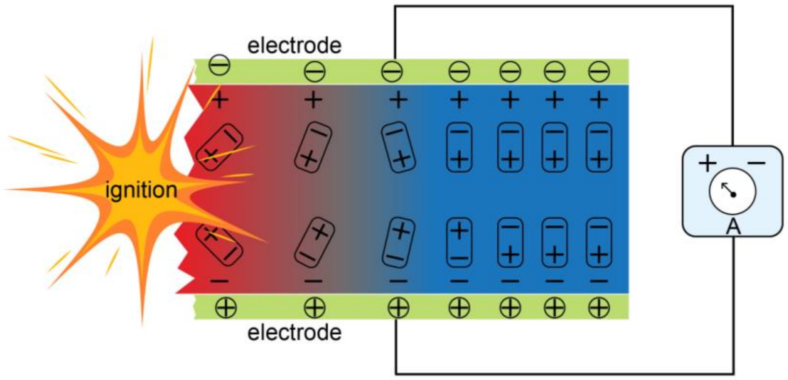

Supplementary Fig. 1. Schematic figure for electricity generation in energetic molecular ferroelectrics.

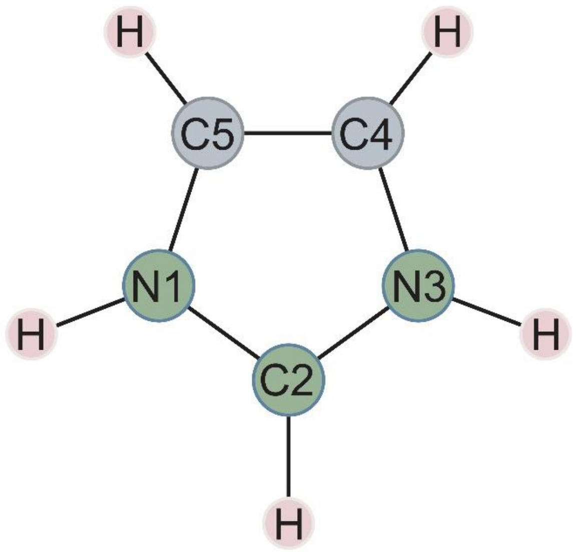

Supplementary Fig. 2. Chemical structures of imidazolium cation.

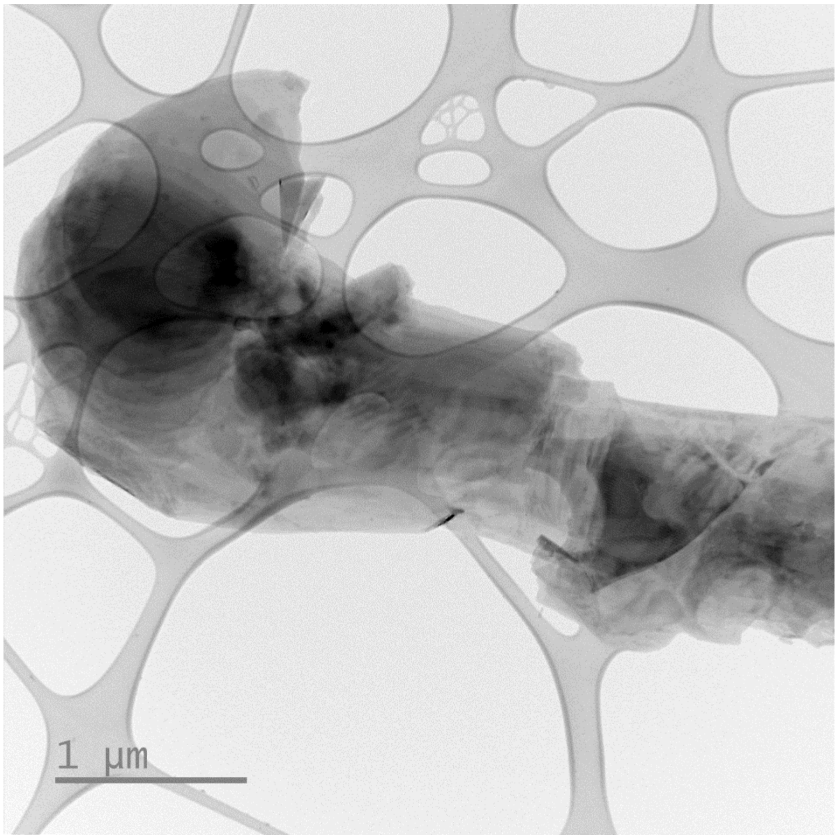

Supplementary Fig. 3. TEM for ferroelectric EIP.

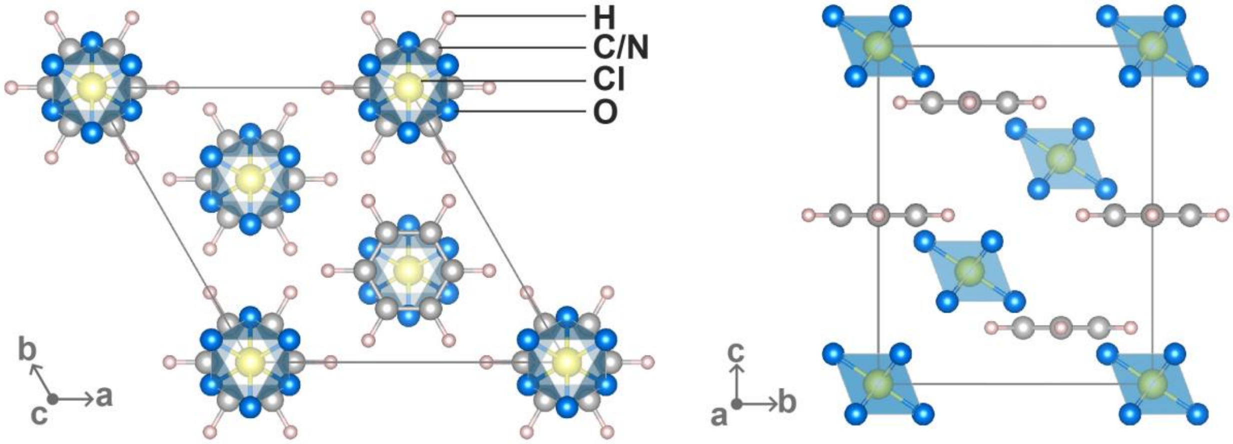

Supplementary Fig. 4. Crystal structure of paraelectric EIP at 385K.

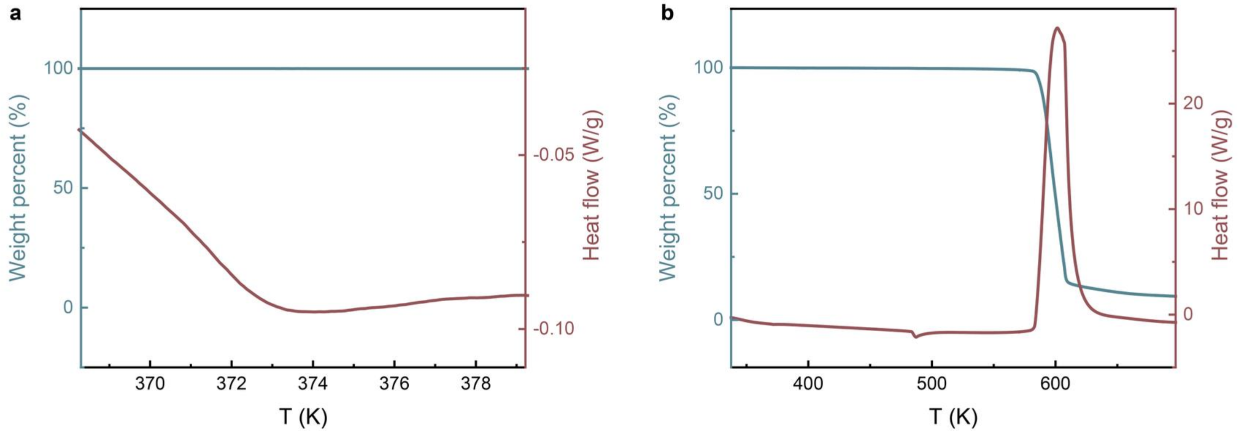

Supplementary Fig. 5. TGA and DSC results of $ImCO_4$ being heated at 10 K minute^{-1} under (a) N_2 and (b) Air. Supplementary Fig. 5a is the zoomed part of Fig. 1e.

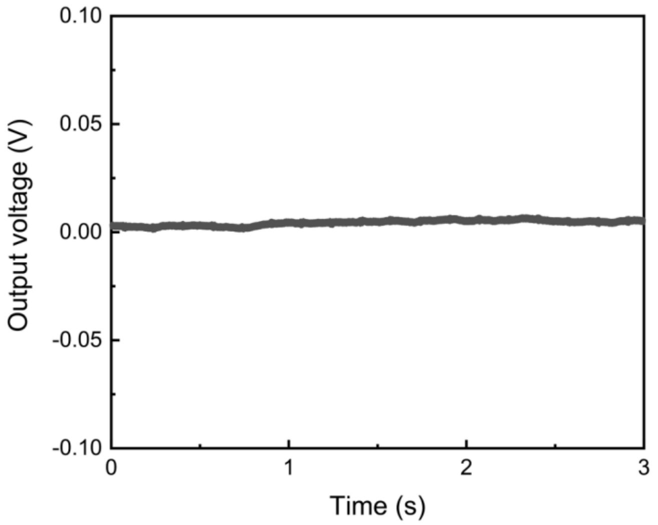

Supplementary Fig. 6. Time dependent output voltage generated by reaction of EIP. In the control experiment, EIP device is kept at 400 K initial environment.

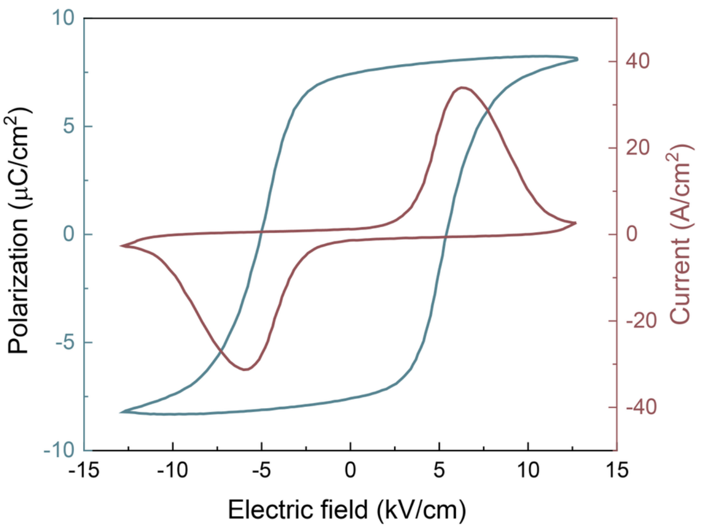

Supplementary Fig. 7. P-E and I-E loops of EIP at room temperature (100 Hz). The maximized current at the coercive field is known as direct evidence of ferroelectricity as the current peak is generated by the dipole reversal rather than electrical conductivity.

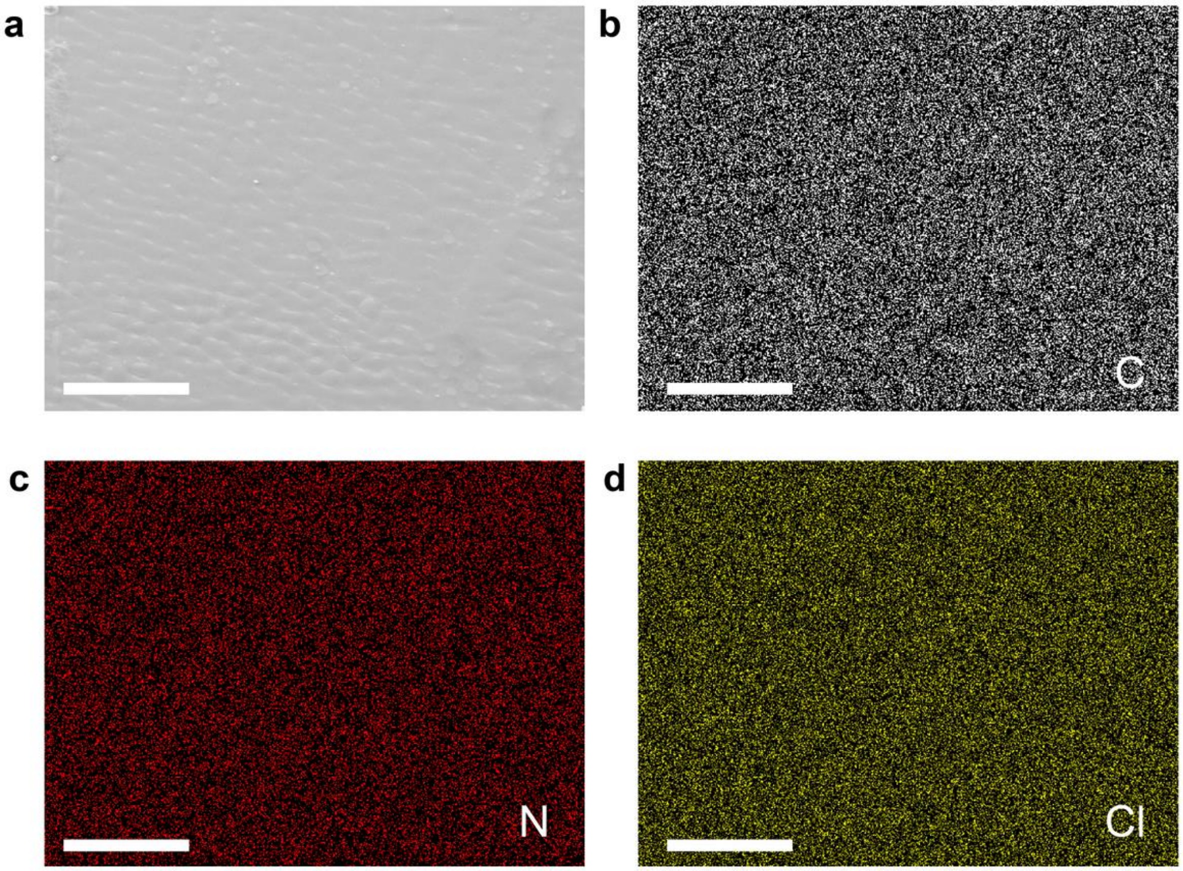

Supplementary Fig. 8. SEM image and EDS mapping of EIP crystal. (a) SEM image and (b)-(d) EDS mapping of C, N and Cl element. The scale bar is 90 μm.

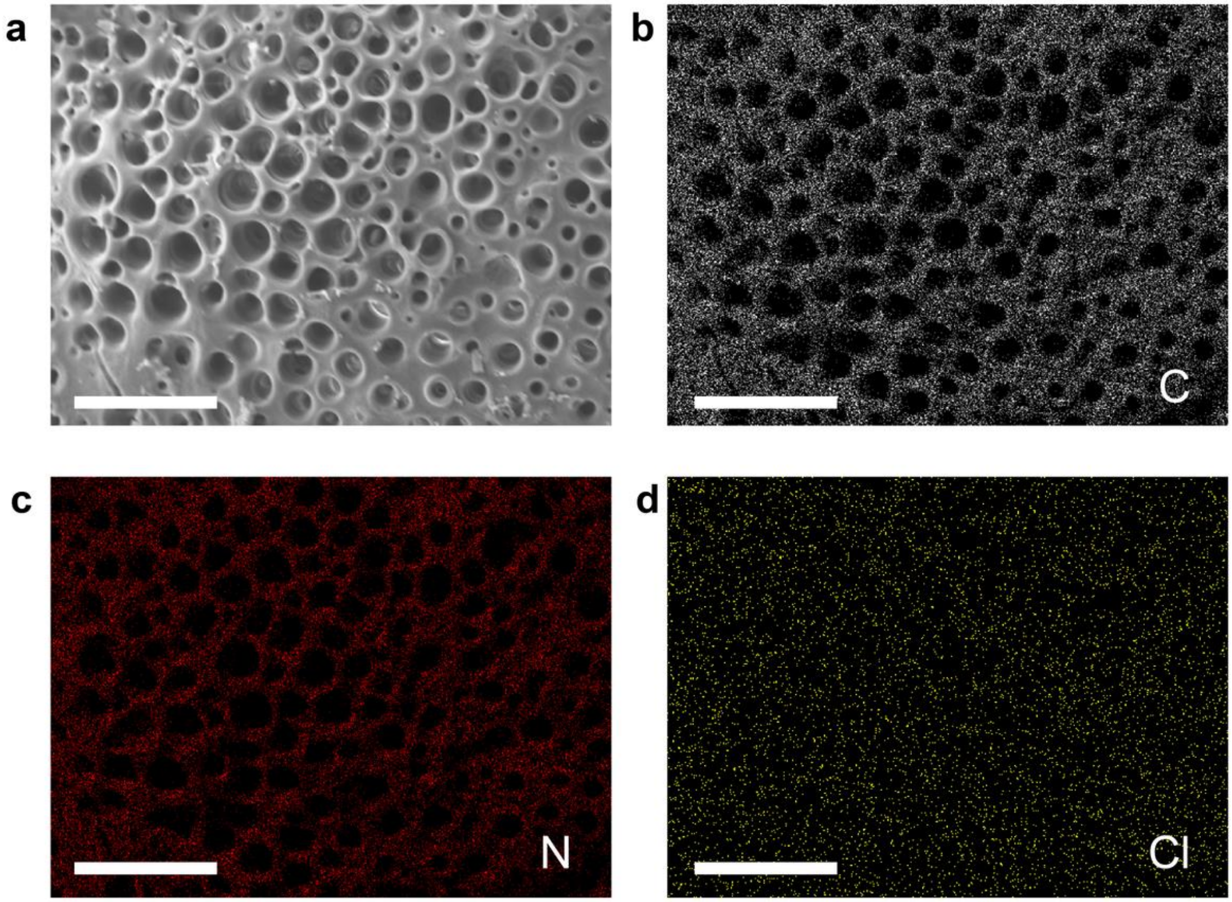

Supplementary Fig. 9. SEM image and EDS mapping of decomposed EIP. (a) SEM image and (b)-(d) EDS mapping of C, N and Cl element. The scale bar is 30 μm.

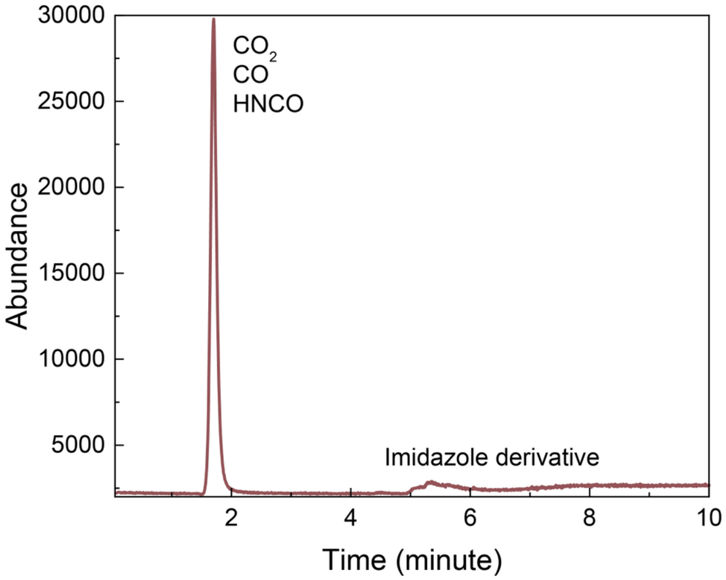

Supplementary Fig. 10. Pyrolysis study for EIP at 623K

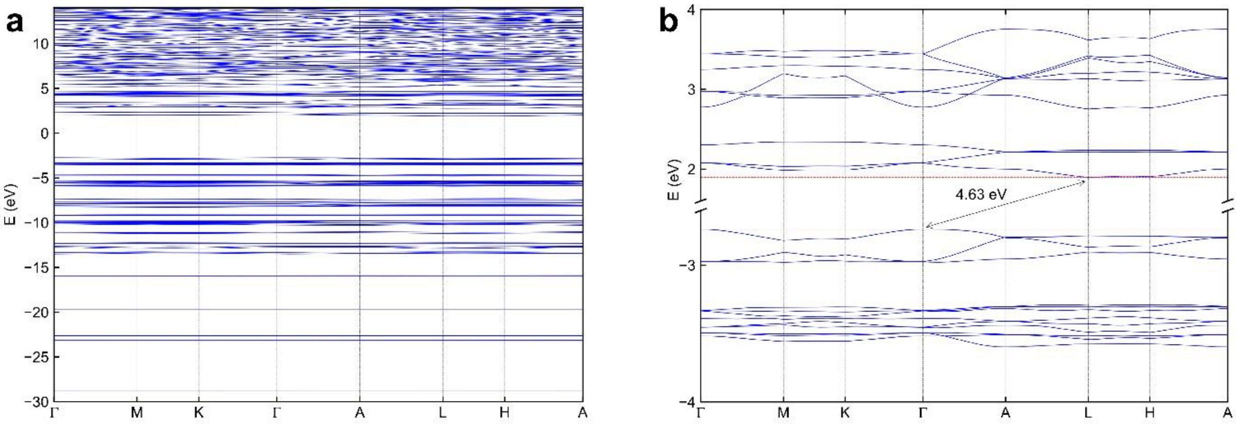

Supplementary Fig. 11. Calculated electronic band structure of EIP (a) electronic band structure (b) zoomed-in -plot for band-gap region

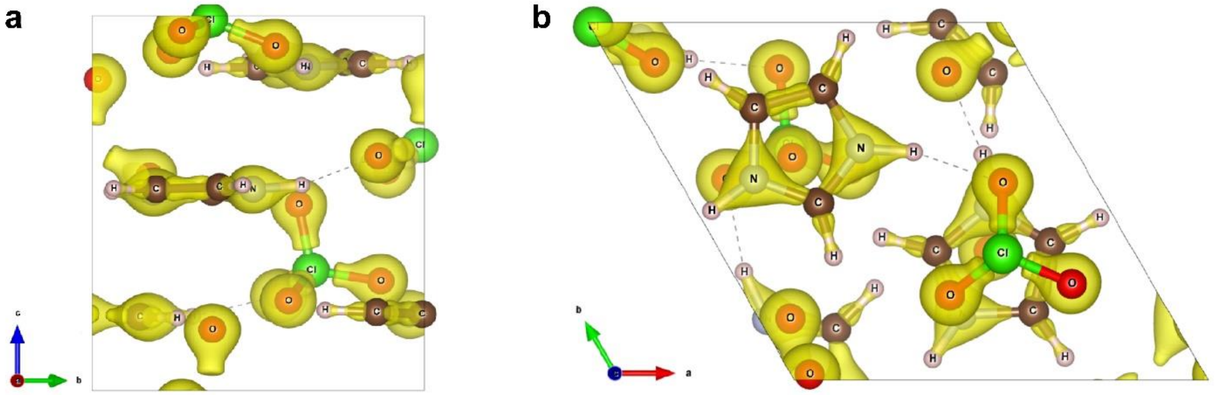

Supplementary Fig. 12. Calculated charge density of EIP. (a) Charge density along the a axis. (b) Charge density along the c axis.

The electronic band structures are determined using the same parameters as in the self-consistent DFT calculation. The structure along the same high-symmetry paths ($\Gamma - M - K - \Gamma - \mathrm{A} - \mathrm{H} - \mathrm{L} - \mathrm{A}$) is shown in Supplementary Fig. 11. The bands are relatively flat, as EIP is an ionic molecular crystal. EIP shows an indirect gap of 4.6 eV. Supplementary Fig. 12. shows the charge density of the structure. Higher densities are noticeable around N and O atoms, showing the large electronegativity of those atoms.

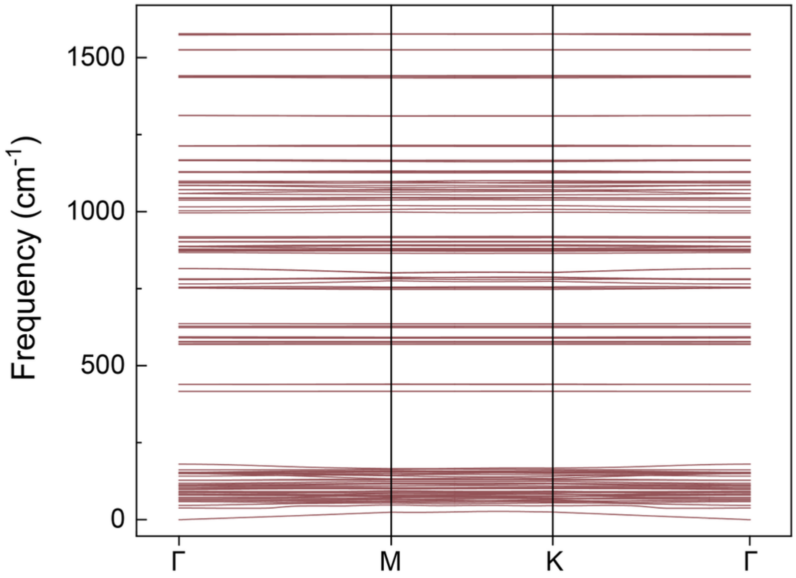

Supplementary Fig. 13. Calculated phonon dispersion for EIP.

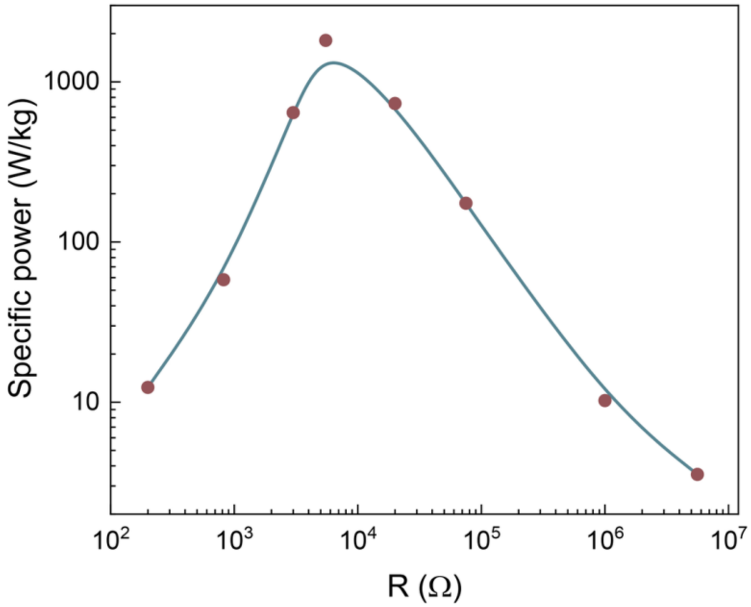

Supplementary Fig. 14. External loading resistance dependence of specific power.

Supplementary Table S2. EDS for EIP and decomposed EIP.

Element	EIP (Atomic%)	decomposed EIP (Atomic%)
C	34.19	44.95
N	23.05	53.13
O	33.52	1.54
Cl	9.24	0.38
Totals	100	100

Cohesive energy

The cohesive energies are reported in two ways, one based on the intramolecular cohesive energy $E_{cohesive}^{intra}$, which is the energy required to separate all atoms, and the second using the intermolecular energy $E_{cohesive}^{inter}$, which is the energy required to separate the molecular crystal into the charged imidazolium and perchlorate molecules.

$$E_{cohesive}^{intra} = \sum E_{atoms} - E_{crystal}$$

$$E_{cohesive}^{inter} = \sum E_{molecule} - E_{crystal}$$

In Supplementary Table S3, we show cohesive energy per unit mass $\frac{E_{cohesive}}{M}$, where M is the mass of the unit cell. For comparison, experimental heat release of EIP in this study, measured heats of formation for crystal and gas-phase imidazole[25], and experimental gas-phase deprotonation enthalpy for perchlorate acid[26] are shown.

Supplementary Table S3. Intermolecular and intramolecular cohesive energy per unit mass of EIP. $\Delta H_{release}^{expt}$ is the heat release of EIP during decomposition at 622K. $\Delta \mathrm{H}_{\mathrm{f}}^{Im}(cr)$ is the heat of formation of crystalline Imidazole. $\Delta \mathrm{H}_{f}^{Im}(g)$ is the heat of formation of gaseous Imidazole. $\Delta \mathrm{H}_{r}^{\mathrm{per}}(g)$ is the gas-phase deprotonation enthalpies.

	$E_{cohesive}^{intra}/M$	$E_{cohesive}^{inter}$ $/M$	$\Delta H_{release}^{expt}$	$\Delta \mathrm{H}_{\mathrm{f}}^{Im}(cr)$	$\Delta \mathrm{H}_{f}^{Im}(g)$	$\Delta \mathrm{H}_{r}^{\mathrm{per}}(g)$
Energy (kJ/kg)	44975.544	3853.664	3066.6	731.5	1952.2	12452.8

Supplementary References

1 Zhang, Y. *et al.* A molecular ferroelectric thin film of imidazolium perchlorate that shows superior electromechanical coupling. *Angew. Chem. Int. Ed.* **53**, 5064-5068 (2014).

2 Luo, E., Xie, Z., Xu, J., Wilson, I. & Zhao, L. In situ observation of the ferroelectric-paraelectric phase transition in a triglycine sulfate single crystal by variable-temperature electrostatic force microscopy. *Phys. Rev. B* **61**, 203 (2000).

3 Liao, W. Q., Tang, Y. Y., Li, P. F., You, Y. M. & Xiong, R. G. Large Piezoelectric Effect in a Lead-Free Molecular Ferroelectric Thin Film. *J. Am. Chem. Soc.* **139**, 18071-18077 (2017).

4 Pan, Q. *et al.* A Molecular Polycrystalline Ferroelectric with Record-High Phase Transition Temperature. *Adv. Mater.* **29** (2017).
5 Tang, Y.-Y. *et al.* Ultrafast polarization switching in a biaxial molecular ferroelectric thin film:[Hdabco]ClO_4. *J. Am. Chem. Soc.* **138**, 15784-15789 (2016).
6 Shi, P.-P., Tang, Y.-Y., Li, P.-F., Ye, H.-Y. & Xiong, R.-G. De novo discovery of [Hdabco] BF4 molecular ferroelectric thin film for nonvolatile low-voltage memories. *J. Am. Chem. Soc.* **139**, 1319-1324 (2017).
7 Pan, Q. *et al.* A Molecular Polycrystalline Ferroelectric with Record-High Phase Transition Temperature. *Adv. Mater.* **29**, 1700831 (2017).
8 Ye, H.-Y. *et al.* Molecular ferroelectric with most equivalent polarization directions induced by the plastic phase transition. *J. Am. Chem. Soc.* **138**, 13175-13178 (2016).
9 You, Y.-M. *et al.* Quinuclidinium salt ferroelectric thin-film with duodecuple-rotational polarization-directions. *Nat. Commun.* **8**, 1-7 (2017).
10 Li, P.-F. *et al.* Anomalously rotary polarization discovered in homochiral organic ferroelectrics. *Nat. Commun.* **7**, 1-9 (2016).
11 Pan, Q. *et al.* A three-dimensional molecular perovskite ferroelectric:(3-ammoniopyrrolidinium) $RbBr_3$. *J. Am. Chem. Soc.* **139**, 3954-3957 (2017).
12 Zhang, W.-Y. *et al.* Precise Molecular Design of High-Tc 3D Organic–Inorganic Perovskite Ferroelectric:[MeHdabco] RbI3 (MeHdabco= N-Methyl-1, 4-diazoniabicyclo [2.2. 2] octane). *J. Am. Chem. Soc.* **139**, 10897-10902 (2017).
13 Xu, W.-J. *et al.* A molecular perovskite with switchable coordination bonds for high-temperature multiaxial ferroelectrics. *J. Am. Chem. Soc.* **139**, 6369-6375 (2017).
14 You, Y.-M. *et al.* An organic-inorganic perovskite ferroelectric with large piezoelectric response. *Science* **357**, 306-309 (2017).
15 Fu, D.-W. *et al.* Diisopropylammonium bromide is a high-temperature molecular ferroelectric crystal. *Science* **339**, 425-428 (2013).
16 Harada, J. *et al.* Plastic/ferroelectric crystals with easily switchable polarization: low-voltage operation, unprecedentedly high pyroelectric performance, and large piezoelectric effect in polycrystalline forms. *J. Am. Chem. Soc.* **141**, 9349-9357 (2019).
17 Ramadan, K. S., Sameoto, D. & Evoy, S. A review of piezoelectric polymers as functional materials for electromechanical transducers. *Smart Mater. Struct.* **23**, 033001 (2014).
18 Yadav, H., Sinha, N., Goel, S., Hussain, A. & Kumar, B. Growth and structural and physical properties of diisopropylammonium bromide molecular single crystals. *J. Appl. Crystallogr.* **49**, 2053-2062 (2016).
19 Harada, J. *et al.* Ferroelectricity and piezoelectricity in free-standing polycrystalline films of plastic crystals. *J. Am. Chem. Soc.* **140**, 346-354 (2018).
20 Li, D. *et al.* Construction of Magnetoelectric Composites with a Large Room-Temperature Magnetoelectric Response through Molecular–Ionic Ferroelectrics. *Adv. Mater.* **30**, 1803716 (2018).
21 Horiuchi, S., Tsutsumi, J. y., Kobayashi, K., Kumai, R. & Ishibashi, S. Piezoelectricity of strongly polarized ferroelectrics in prototropic organic crystals. *J. Mater. Chem. C* **6**, 4714-4719 (2018).
22 Horiuchi, S., Kumai, R. & Tokura, Y. Hydrogen-bonding molecular chains for high-temperature ferroelectricity. *Adv. Mater.* **23**, 2098-2103 (2011).
23 Horiuchi, S. *et al.* Above-room-temperature ferroelectricity and antiferroelectricity in benzimidazoles. *Nat. Commun.* **3**, 1-6 (2012).

24 Pandian, M. S., Ramasamy, P. & Kumar, B. A comparative study of ferroelectric triglycine sulfate (TGS) crystals grown by conventional slow evaporation and unidirectional method. *Mater. Res. Bull.* **47**, 1587-1597 (2012).

25 Jimenez, P., Roux, M., Turrion, C. & Gomis, F. Thermochemical properties of N-heterocyclic compounds I. Enthalpies of combustion, vapour pressures and enthalpies of sublimation, and enthalpies of formation of pyrazole, imidazole, indazole, and benzimidazole. *J. Chem. Thermodyn.* **19**, 985-992 (1987).

26 Meyer, M. M. & Kass, S. R. Experimental and Theoretical Gas-Phase Acidities, Bond Dissociation Energies, and Heats of Formation of HClOx, x=1−4. *J. Phys. Chem. A* **114**, 4086-4092 (2010).
